# Children’s Creativity and Personal Adaptation Resources

**DOI:** 10.3390/bs10020049

**Published:** 2020-02-03

**Authors:** Vera G. Gryazeva-Dobshinskaya, Yulia A. Dmitrieva, Svetlana Yu. Korobova, Vera A. Glukhova

**Affiliations:** Laboratory of Psychology and Psychophysiology of Stress-Resistance and Creativity, Scientific and Educational Center “Biomedical Technologies”, Higher Medical and Biological School, South Ural State University, 454080 Chelyabinsk, Russia; vdobshinya@mail.ru (V.G.G.-D.); k.svetlana-1991@mail.ru (S.Y.K.); gluhova-vera@mail.ru (V.A.G.)

**Keywords:** creativity, children’s personal adaptation resources, intelligence, the Wechsler Intelligence Scale, the E. Torrance Test of Creative Thinking, the G. Rorschach Test

## Abstract

The study provides insights into the aspects of creativity, the structure of psychometric intelligence, and personal adaptation resources of senior preschool children. Creativity and intelligence are presented as general adaptation resources. Existing studies of creative ability and creativity as integral individual characteristics in the context of adaptation are analyzed. The aim is to identify varied sets of creativity and personal adaptation resource markers that differentiate groups of children in order to determine possible strategies for adaptation, preservation, and development of their creative abilities at the beginning of lyceum schooling. It embraces the use of the E. Torrance Test of Creative Thinking (TTCT) (figural version), the Wechsler Intelligence Scale for Children (WISC), and the G. Rorschach Test. A sample of the study consisted of 122 children, aged 6–7 and enrolled in a school. The average IQ score among the children was above 115 (M = 133.7, σ = 9.9). The entire sample was divided into four groups by the originality-elaboration ratio according to the TTCT. The correctness of the children’s division into the groups according to the markers of creativity and personal adaptation resources is confirmed by the discriminant analysis. We have identified the factor structure of creativity, intelligence, and personal adaptation resources in the entire sample of children and in each of the groups. In the group of preschoolers with high originality and elaboration, the resulting structure integrated the components of creativity with personal adaptation resources and intelligence scores. In the group of children with a low level of originality and elaboration, the markers of creativity, intelligence, and personal adaptation resources are not interlinked.

## 1. Introduction

The growing frequency and cultural diversity of contemporary creativity studies are connected with the increase in their importance for social progress [1,2]. Today’s public demand for mass creativity involves promoting the development and preservation of creative potential since childhood and across life span. Moreover, development of creative potential as a complex system depends on the conditions of adaptation in the society at different stages of ontogenesis. People with high creative abilities, who adapt well in new communities, will be successful in unleashing their potential and developing. Those who, having a high creative potential, fail to adapt well, will suffer problems in self-fulfillment and self-promotion [3,4]. Therefore, due to the complex nature of creativity it is important to discern resources that help with such adaptation from those that obstruct it.

This paper focuses on the personal adaptation resources of children with high intelligence scores and various markers of creativity prior to their enrollment in a lyceum school.

The period of schooling is decisive in terms of building creative abilities and adaptation resources in individuals. Schooling nurtures socio-cultural standards of activity and communication. Thus, a conflict is possible between a creative potential and initiative and the need to comply with standards [5]. The specific problem of adaptation in creative personalities is revealed in childhood, from kindergarten to school, when children’s adaptive activity grows and creativity drops [5,6,7].

Adaptability in this study is understood as a structural feature of the target future action of subjects and includes personal components that determine its development: Impulse and reflection of forces, desire and anticipation, aspirations and self-confidence, perseverance and performance, forecast of one’s own capabilities and the capabilities of the environment, and situation [8].

Aim: To identify varied sets of creativity and personal adaptation resource markers that differentiate groups of children in order to determine possible strategies for adaptation, preservation, and development of their creative abilities at the beginning of lyceum schooling.

To study “creativity in the context of adaptation”, it is imperative to identify the differentiating grounds according to creativity metrics, identify options for combining them with the adaptation resources of preschoolers, and choose activities with a high level of uncertainty so that a child can independently demonstrate various forms of creativity and adaptability.

## 2. Literature Review

### 2.1. Creativity and Intelligence as General Adaptation Resource

The adaptation processes are explored in the context of intelligence and its development in ontogenesis [9,10]. Hence, we analyzed scientific studies of adaptation resources of children with different scores of intelligence and creativity.

The ratio of intelligence and adaptation is complex and variable due to diverse markers of personal adaptation to the world. The adaptation markers include study progress and professional achievements—“subject-symbolic adaptation”, adaptation to the social environment—“social adaptation”, and coping behavior as harmonization of the inner world—“personal adaptation” [11]. It is revealed that adaptation difficulties more often affect children with higher and lower intelligence scores compared with children with mean intellectual scores [11], as well as with higher and lower creativity [12].

For the study of “creativity in the context of adaptation”, the findings of researching the creativity-intelligence ratio as an adaptation resource are crucial. Some studies identify the creativity-intelligence ratio [13,14]; others reject any link, citing them as different types of abilities, their ratio depending on the “intellectual range” [10,15].

J.-H. Guignard et al. found that high verbal ability children showed significantly higher scores on verbal creative potential [16]. The complex relationships of verbal and non-verbal intelligence with verbal and non-verbal creativity are revealed in the study of situational and interpersonal determinants of the manifestation of intelligence and creativity [17].

E. Torrance’s studies revealed that the creativity-intelligence interrelationship does not exist in all samples of subjects. According to the intellectual threshold theory, when IQ is below 115–120, intelligence and creativity form a single factor, with IQ above 120 they are independent factors [18]. B. Shi’s et al. studies revealed that the breakpoint of the relation between creativity and intelligence occurred at an IQ of 110.1 [19].

### 2.2. Structure of Creative Abilities in Adaptational Context

Let us review the concepts and approaches to insights into creativity, which identify its key features, which are significant for the differentiation of subject groups.

The core approach to the study of the structure of creative abilities is the psychometric approach developed by J. Guilford and E. Torrance [13,18,20,21]. The key markers of creativity offered by J. Guilford were modified by E. Torrance and included fluency, flexibility, originality, and elaboration [20,22,23]. The analysis of creativity research reveals its standard of definition by two criteria—originality (novelty of ideas) and applicability (corresponding to reality), while creativity metrics can include various tasks: cognitive, divergent thinking, problem thinking, etc. [24].

Creativity is measured using the widely popular E. Torrance Test of Creative Thinking (TTCT). Ample studies have confirmed the good psychometrics of the test—high validity and reliability [7,18,25]. The adaptation and standardization of the TTCT was carried out on various samples [22,26,27].

According to E. Torrance, the key criteria differentiating subjects by creativity are scores of originality as the ability to create new things and elaboration as the ability to detail and deepen initial ideas, which increases their complexity and improves presentation. It has been revealed that children with a high level of originality excel in curiosity and independence, and are prone to take risks, while children with a high level of elaboration are distinguished by the desire to have the upper hand and are emotionally insensitive [23]. 

The studies of the factor structure of creativity by the TTCT revealed sets of variables featuring the innovation and adaptation potentials of subjects, the relationship of creativity with such personal qualities as openness to new things, curiosity, tolerance for uncertainty, and risk acceptance [28,29].

In addition, studies revealed two-factor structure of variables (innovation factor—originality and fluency; adaptation factor—elaboration and abstractness) in the context of the Adaption-Innovation Theory [30,31].

To summarize, aforementioned analysis allows us to differentiate our samples by two variables: originality and elaboration. Those two variables, representing both the potential to create new and adaptational potential, are abilities that are required to bring something new into being.

### 2.3. Creativity as Integrative Individual Characteristic in Adaptational Context

Another approach to the study of creativity is integrative: creativity is cited as a systemic feature, including intelligence, knowledge, mindset, individual traits, motivation, and environment [32]. M. Csikszentmihalyi and R. Robinson consider creativity as a combination of opposing personal features and systemic quality that change over time and depend on environmental factors [33].

In the studies of creativity as systemic quality, personal components were identified—intrinsic motivation [34], intellectual initiative [35], supra-situational activity [8], and socio-psychological characteristics—status in class and adoption of identity [6]. The issue of ‘creativity and adaptation’ was explored in the context of studying creativity markers as predictors of academic progress; the specifics of teaching children with different intellectual and creative abilities [27,36,37]. 

In different age samples, the personal properties that manifest themselves in a situation of uncertainty (according to the G. Rorschach Test) were investigated; properties aimed at changing and creativity, and properties aimed at stabilizing and maintaining standards. The first group of properties includes intellectual initiative, flexibility, composite thinking, originality, and perception, while second one includes intellectual control, emotional reactivity, anxiety, and realistic perception [38,39].

Based on the theoretical analysis, creativity markers were chosen as grounds for differentiating groups of children—originality and elaboration taking into account intelligence scores (according to E. Torrance). For the study of children’s personal adaptation resources, opposite personality traits were selected, including types of experiences (the ratio of introversion and extratension), emotional reactivity, intellectual initiative and intellectual control, composite thinking, realistic perception, and originality, providing both the creativity and adaptability in situations of uncertainty (according to G. Rorschach).

Hypothesis: Children with high intelligence scores who have different levels of manifested creative abilities—originality and elaboration—feature specific sets of personal adaptation resources.

## 3. Materials and Methods

### 3.1. Design of Research

The empirical study consisted of the psychological diagnostic of children using three methodics: The Wechsler Intelligence Scale for Children (WISC), the E. Torrance Test of Creative Thinking, and the G. Rorschach Test.

For the study of creativity, intelligence, and personal adaptation resources, discriminant and factor analysis of captured psychological screening results were applied. The analysis as a whole covered the entire sample and groups of children with different creativity scores (originality and elaboration). All calculations were performed using the IBM SPSS Statistics package.

All subjects gave their informed consent for inclusion before they participated in the study. The study was conducted in accordance with the Declaration of Helsinki, and the protocol was approved by the Local Ethics Committee of the Higher Biomedical School South Ural State University of 307/20 of 5 December 2019.

### 3.2. Sample

The study involved 122 children aged 6–7, 65 boys and 57 girls, enrolled in a school in Chelyabinsk. The sample consisted of children who belonged to the same ethnic group—Russians who live in Chelyabinsk city. Later, all these children were accepted in different classes of the same school.

The IQ average scores among the children was above 115 (M = 133.7, σ = 9.9). The sample was divided into four groups by originality (M = 26.3, σ = 13.7) and elaboration ratio (M = 63.9, σ = 32.3). The first group was 25 children with above average originality and elaboration scores. The second group was 22 children with above average originality and below average elaboration. The third group was 25 children with below average originality and above average elaboration. The fourth group was 50 children with below average originality and elaboration.

### 3.3. Methods

For the psychological assessment of creativity and its key markers, we have used the TTCT; the figural version adapted by E.E. Tunik [22,23]. The test embraces four independent metrics: “fluency”, “flexibility”, “originality”, and “elaboration” measuring imaginative creativity and the overall creativity as the sum of four independent indicators (ΣCr). “Fluency” characterizes the ability of subjects to generate ample diverse ideas. “Flexibility” describes the potential of subjects to offer multidirectional ideas and apply a variety of strategies. “Originality” characterizes the skills of subjects to generate non-standard ideas. “Elaboration” reflects the ability of subjects to elaborate on the emerging ideas.

For the psychological screening of intellectual levels, we used the Wechsler Intelligence Scale for Children (WISC) with five verbal subtests (Information, Comprehension, Arithmetic, Similarities, Vocabulary;) and five non-verbal subtests (Picture Completion, Picture Arrangement, Block Design, Object Assembly, Coding). In this study, we used the markers of verbal (VIQ) and non-verbal (PIQ) intelligence and a general IQ score.

Average results of intelligence, creativity, and adaptive personal resources in groups are shown in Table 1. 

For the psychological screening of creative and adaptive resources, the G. Rorschach Test was applied [40,41,42]. To measure personal resources in situations of uncertainty, the most valid and reliable indicators of the Rorschach Test are: The amount of experience (∑M∑C), the intellectual control (∑F), intellectual initiative (∑M), emotional reactivity (∑C, including C—impulsivity, CF—lability of affect, sensitivity, suggestibility, FC—intellectual control of emotional reactivity, ability to adapt); psychophysical activity (ΣFMm), compositional thinking (Z), сommonality of interpretation (Pop—index of realism), originality of interpretations (Or—originality index) [38].

The validity of the Rorschach Test results and their combinations were confirmed in multiple scientific studies [41,42,43,44,45], including D.P. Ogdon’s review of the Rorschach Test’s validity on different samples [46].

## 4. Results

### 4.1. Differentiation of Preschoolers with Different Originality and Elaboration Scores

The differentiation of children according to the originality-elaboration ratio was tested using discriminant analysis. The captured findings of assessing the correctness of classification and assignment of subjects into groups, the analysis of the contributions of creativity, and personal adaptation resources to the core discriminant function are shown in Table 2.

The greatest contribution to the core discriminant function, which is a linear equation for classifying children with different creativity scores, is made by the variables: Originality, elaboration, and overall creativity (the TTCT), and compositional thinking (the G. Rorschach Test).

### 4.2. Structure of Children’s Creativity, Intelligence and Personal Adaptation Resources 

The structure of children’s creativity, intelligence, and personal adaptation resources was assessed on the basis of the factor analysis of captured psychological screening findings (Table 3, Table 4 and Table 5).

The factor structure of creativity, intelligence, and personal resources throughout the sample forms five factors (77.62% of explainable variance), in which variables of methods are not included in common factors, while personal resources are represented by two factors (with 18.8% and 16.9% variance). Creativity is represented by two factors (with 17.4% and 11.2% variance) and intelligence by one factor (13.3% variance) in general; in a sample of children with IQ scores above the norm, the factor structure of creativity, intelligence, and personal resources is represented by orthogonal factors, which indicates the unrelated nature of these phenomena.

In Group 2, children with above average originality and below average elaboration, the first set of variables is a bipolar factor formed by several features of personal resources (amount of experience, emotional reactivity, originality of interpretations) and IQ at one pole and originality according to the TTCT at the other pole. The second set of variables includes only the markers of personal adaptation resources—indicators of intellectual control, “realistic” perception, and psychophysical activity. The third set of variables embraces such features of creativity as fluency and flexibility, as well as indicators of intelligence: total IQ and verbal intelligence (VIQ). The fifth set of variables includes flexibility, total IQ, and non-verbal intelligence (PIQ). The fourth set of variables is represented by overall creativity and elaboration and personal resources; intellectual initiative and compositional thinking (Table 4). Thus, children with a high level of originality have more pronounced interrelations of creativity and intelligence, as well as creativity and those personal resources that support both intellectual activity and creativity, such as intellectual initiative and complexity of interpretations.

In Group 3, children below average originality and above average elaboration, the first set of variables is a bipolar factor formed by several features of personal resources. At one pole of the factor, there are the amounts of experience, emotional reactivity, compositional thinking, and originality of perception, and at the other pole—standard, realistic perception. The second set of variables also represents the bipolar factor: One pole includes features of creativity—overall creativity, flexibility, and originality, and the other pole embraces the indicators of personal adaptation resources—intellectual initiative, psychophysical activity, and realistic perception. The third set of variables covers all IQs: Total IQ score, VIQ, and PIQ, and fluency as a metric of creativity. The fourth set of variables includes overall creativity and elaboration as well as intellectual control and originality of perception (Table 5). Thus, for children with a high level of elaboration, a significantly lower integration of creativity with personal resources is typical; there is no integration with emotional resources and there is a negative relationship with personal resources of activity. The overall creativity and its development metric is supported by personal adaptation resources—intellectual control.

In Group 4, children demonstrating below average originality and elaboration, the markers of creativity, intelligence, and personal adaptation resources are independent and form four orthogonal factors. The first set of variables is formed only by the metrics of personal resources (and does not include only one indicator—standardity, realistic perception). The second set of variables is formed only by creativity markers. The third set of variables includes the markers of intelligence and its components. Only the fourth set of variables embraces two features of creativity; the overall creativity and elaboration, and the indicator of personal adaptation resources—intellectual control. Thus, as in the whole sample of children with below average originality and elaboration, the markers of creativity, intelligence, and personal adaptation resources are independent.

## 5. Discussion and Conclusions

Children with high levels of intellectual abilities differentiated by key characteristics of creativity—originality and elaboration—differ, in general, by characteristics of creativity and personal adaptation resources (confirmed by discriminant analysis). The maximum contribution to the proposed classification is made by the indicators of originality, elaboration, overall creativity, and compositional thinking in a situation of uncertainty.

In general, in a sample of children with a high level of intellectual abilities, the factor structure of the characteristics of creativity, intelligence, and personal resources is represented by orthogonal factors, showing the unrelated nature of these phenomena. This helps to confirm the “threshold theory” regarding intelligence proposed by E. Torrance. However, in the group of children with high levels of originality and elaboration, and in the group of children with high level of originality, sets of variables were captured, including both characteristics of intelligence and creativity.

The factor structure of creativity, intelligence, and personal adaptation resources in each of the four groups of children demonstrating various levels of originality and elaboration has been determined. In the group of preschoolers with a high level of intellectual abilities, originality, and elaboration, the resulting structure is as integrated as possible; creativity is simultaneously linked to personal adaptation resources, and to overall intelligence and its components (verbal and non-verbal intelligence). Creativity, intelligence, and adaptation resources in the group of children with a high level of intellectual abilities and low levels of originality and elaboration are not connected at all. 

The most problematic from the point of view of personal adaptation resources are two groups of children. This is the group of children with a high level of intellectual abilities and a high level of originality characterized by interrelations of creativity and intelligence, as well as creativity and those personal resources that support both intellectual activity and creativity—intellectual initiative and complexity of interpretations. It is this complexity of properties that is decisive in the effective strategy of school adaptation, aimed at supporting “innovative creativity” [7,47]. Another group of children with a high level of intellectual abilities and high level of elaboration, featuring a much poorer integration of creativity with personal resources, have a negative relationship with personal resources of activity. These children’s overall creativity and its elaboration indicator is supported by a quite effective personal adaptation resource—intellectual control. These properties most likely determine the strategy of adaptation, aimed at supporting “adaptive creativity” [7,28,47].

In general, the study revealed the possible foundations for a differentiated approach to the adaptation of children with high intellectual and creative abilities prior to the start of schooling.

## 6. Limitations

The first limitation is the sample that is shifted by intelligence scores: The mean IQ is 133.7. The second limitation of the sample is that the children included in this study were preparing for the enrolment in a lyceum school with high levels of development. The third limitation is the use of the TTCT to screen the characteristics of creativity. The research findings could be improved through the simultaneous combination of several metrics of creativity and real creative achievements.

## Figures and Tables

**Table 1:** Average results of intelligence, creativity, and adaptive personal resources in groups.

Sample	Total Score	E. Torrance Test of Creative Thinking (TTCT)				Rorschach Test				
	IQ	Fluency	Flex	Orig	Elab	ΣMΣC	ΣC	ΣM	ΣF	Z
Group 1	135.0 ± 11.4	25.6 ± 7.2	18.0 ± 4.7	39.5 ± 10.5	98.2 ± 32.0	9.0 ± 5.1	7.0 ± 4.0	2.0 ± 2.2	21.8 ± 6.9	6.0 ± 4.9
Group 2	130.9 ± 9.2	24.9 ± 5.8	17.1 ± 3.8	40.3 ± 12.7	42.0 ± 14.3	8.0 ± 5.9	6.2 ± 5.5	1.8 ± 2.0	17.4 ± 9.4	6.8 ± 4.8
Group 3	134.4 ± 10.0	15.6 ± 4.9	11.3 ± 3.0	17.4 ± 6.0	90.5 ± 21.5	9.0 ± 6.6	7.1 ± 6.3	1.9 ± 1.6	19.3 ± 8.5	6.9 ± 3.7
Group 4	132.4 ± 11.0	16.7 ± 5.2	12.6 ± 3.5	17.7 ± 4.8	43.4 ± 12.3	5.9 ± 3.7	4.5 ± 3.3	1.3 ± 1.6	15.6 ± 9.0	3.9 ± 3.2

IQ—general score of intellectual level according to the Wechsler Intelligence Scale for Children (WISC); Flex—flexibility according to the TTCT, Orig—originality according to the TTCT, Elab—elaboration according to the TTCT; ∑M∑C—the amount of experience, ∑C—emotional reactivity, ∑M—intellectual initiative, ∑F—the intellectual control, Z—compositional thinking according to the G. Rorschach Test.

**Table 2:** Findings of discriminant analysis.

Basis for Classification	Correctness of Classification, %	Core Discriminant Function
Originality-elaboration ratio	92.0	F = −4.891 + 0.071 × Orig + 0.023 × Elab + 0.012 × ΣCr + 0.002 × Z

Orig—originality according to the TTCT, Elab—elaboration according to the TTCT, ΣCr—overall creativity according the TTCT, Z—compositional thinking according to the G. Rorschach Test.

**Table 3:** Structure of children’s creativity, intelligence, and personal resources, Group 1.

Variables	Factors and Factor Load Variables			
	1	2	3	4
Creativity (TTCT)				
Overall creativity (Σ according to the test findings)	0.466	0.419		0.583
Fluency		0.900		
Flexibility		0.803		
Originality		0.933		
Elaboration	0.445			0.549
Intelligence (WISC)				
IQ			0.817	0.426
Verbal intelligence (VIQ)			0.888	
Non-verbal intelligence (PIQ)				0.799
Personal resources (Rorschach Test)				
Amount of experience (ΣMΣC)	0.950			
Intellectual control (ΣF)	0.793			
Emotional reactivity (ΣC)	0.812			
Intellectual initiative (ΣM)	0.731			
Psychophysical activity (ΣFMm)	0.775			
Compositional thinking (Z)	0.773			
Originality of interpretations (Or)	0.812			
Commonality of interpretations (Pop)	0.471	0.496		0.493
Variance	33.72%	19.96%	13.69%	12.66%

**Table 4:** Structure of children’s creativity, intelligence, and personal resources, Group 2.

Variables	Factors and Factor Load Variables				
	1	2	3	4	5
Creativity (TTCT)					
Overall creativity (Σ according to the test findings)			0.481	0.689	
Fluency			0.739		
Flexibility			0.802		0.478
Originality	−0.407				
Elaboration				0.724	
Intelligence (WISC)					
IQ	0.547		0.549		0.560
VIQ (verbal intelligence)			0.710		
PIQ (non-verbal intelligence)					0.893
Personal resources (Rorschach Test)					
Amount of experience (ΣMΣC)	0.943				
Intellectual control (ΣF)	0.440	0.836			
Emotional reactivity (ΣC)	0.917				
Intellectual initiative (ΣM)				0.705	
Psychophysical activity (ΣFMm)		0.842			
Compositional thinking (Z)	0.548	0.419		0.436	
Originality of interpretations (Or)	0.614	0.433			
Commonality of interpretations (Pop)		0.903			
Variance	23.76%	18.10%	16.63%	12.89%	10.05%

**Table 5:** Structure of children’s creativity, intelligence, and personal resources, Group 3.

Variables	Factors and Factor Load Variables			
	1	2	3	4
Creativity (TTCT)				
Overall creativity (Σ according to the test findings)		0.421		0.801
Fluency			0.490	
Flexibility		0.609		
Originality		0.796		
Elaboration				0.869
Intelligence (WISC)				
IQ			0.972	
Verbal intelligence (VIQ)			0.765	
Non-verbal intelligence (PIQ)			0.679	
Personal resources (Rorschach Test)				
Amount of experience (ΣMΣC)	0.936			
Intellectual control (ΣF)				0.667
Emotional reactivity (ΣC)	0.893			
Intellectual initiative (ΣM)		−0.490		
Psychophysical activity (ΣFMm)		−0.708		
Compositional thinking (Z)	0.796			
Originality of interpretations (Or)	0.689			0.437
Commonality of interpretations (Pop)	−0.431	−0.653		
Variance	21.08%	16.94%	15.96%	15.43%
